# Characterization of atypical acute promyelocytic leukaemia: Three cases report and literature review

**DOI:** 10.1097/MD.0000000000015537

**Published:** 2019-05-13

**Authors:** Xiaoxue Wang, Jing Wang, Lijun Zhang

**Affiliations:** Department of Hematology, The First Hospital, China Medical University, Shenyang , China.

**Keywords:** acute promyelocytic leukemia, chemotherapy, cytogenetics, molecular biology

## Abstract

**Rationale:**

The vast majority of acute promyelocytic leukemia (APL) is characterized with a specific chromosomal translocation t (15, 17) (q22, q21), which fuses PML-RARα leading to a good response to all-trans retinoic acid (ATRA) and arsenic trioxide (ATO). However, there are few cases of atypical APL, including PLZF-RARα, F1P1L1-RARα, STAT5b-RARα, et al. Neither PLZF-RARα nor STAT5b-RARα are sensitive to ATRA and ATO, and the prognosis is poor.

**Patient concerns:**

Here we have 3 cases (PLZF-RARα, n = 2; STAT5b-RARα, n = 1). Case A, A 53-year-old Chinese female had suffered ecchymosis in both legs for 3 days. Case B, A 44 years old male suffered pain from lower limbs and hip. Case C, 52-year-old male patient presented with fever for 3 weeks invalid to antibiotics and gingival bleeding for 1 week.

**Diagnoses:**

With RT-PCR and karyotype, Case A is diagnosed with STAT5b-RARα-positive APL.

Case B, C are diagnosed with PLZF-RARα-positive APL.

**Interventions:**

In case A, ATO, and ATRA were used for induction treatment. In Case B, ATO, and chemotherapy with DA were given in the first induction treatment. In Case C, ATRA, and ATO were used immediately, subsequently, chemotherapy was added with DA, ATRA, and CAG combination treatment, and medium-dose cytarabine with daunorubicin were given regularly.

**Outcomes:**

In Case A, the patient refused the following treatment and discharged on day 25. In Case B, the patient got the disseminated intravascular coagulation (DIC).In Case C, the patient has survived for 7 months and remains CR.

**Lessons:**

Both STAT5b-RARα-positive APL and PLZF-RARα-positive APL appear to be resistant to both ATRA and ATO, so combined chemotherapy and allo-HSCT should be considered. Since the prognosis and long-term outcome are poor, more clinical trials, and researches should be taken.

## 1 Introduction

The vast majority(>95%) of patients with acute promyelocytic leukemia (APL) are characterized with a specific chromosomal translocation t(15,17)(q22,q21), which fuses the promyelocytic leukemia (*PML*) gene located on chromosome 15 to the retinoic acid receptor a(*RARα*) gene located on chromosome 17.^[1,2]^ So far, typical APL with PML-RARα has responded well to all-trans retinoic acid (ATRA) and arsenic trioxide (ATO). The complete remission rate can achieve 90% and nearly 70% of them are potentially cured.^[3–5]^ Seven types of atypical APL have been found, including PLZF-RARα (promyelocytic leukemia zinc finger), NuMA-RARα(nuclear mitotic apparatus protein),NPM-RARα(nucleophosmin), F1P1L1/-RARα(FIP1-like 1), BCOR-RARα(BCL6 corepressor), STAT5b-RARα(Signal transducer and activator of transcription 5b), and PRKAR1A-RARα(protein kinase A regulatory subunit type 1A). All of the 6 atypical APL have corresponding chromosome translocation as follows: t(11,17) (q23,q21), t(11,17)(q13,q21), t(5,17)(q35,q21), t(4,17)(q12,q21), t(X,17)(p11,q21), and 17q.^[1,6–9]^ We have known that PML-RARα, NuMA-RARα, NPM-RARα are sensitive to ATRA, but both PLZF-RARα and STAT5b-RARα are insensitive.^[7,10]^ Here we will report 3 cases of atypical APL with the clinical feature, treatment and the outcome.

## 2 Cases report

**Figure 1:**
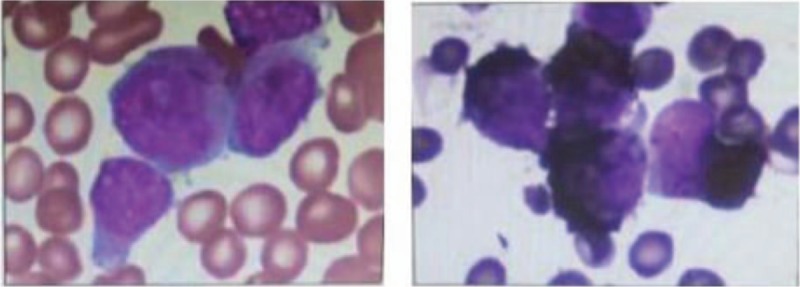
Bone marrow of case A(STAT5b/RARα).

**Figure 2:**
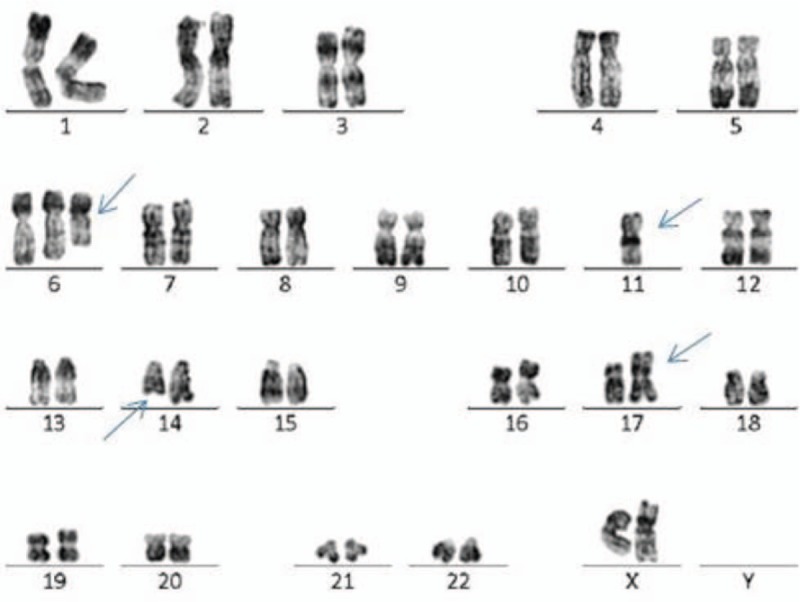
Karyotype of case A 46, XX,+6q-,-11,14q-,?i(17)(q10)/46,XX.

*Case A* A 53-year-old Chinese female who had suffered ecchymosis in both legs for 3 days was admitted to our hospital on June, 2017. Pancytopenia was detected in complete blood count (CBC). Bone marrow (BM) aspirate revealed predominant blasts (Fig. 1). Flow cytometry on the aspirate showed mostly positivity for CD33, CD117, CD34, CD13, MPO, CD64, and CD9, as well as partly positivity for CD123,CD99. Reverse transcription–polymerase chain reaction (RT-PCR) analysis detected STAT5b-RARα fusion transcripts. A routine chromosomal analysis was performed. An abnormal female karyotype 46, XX,+6q-,-11,14q-,?i(17)(q10); 46, XX was detected (Fig. 2). ATO and ATRA were used for induction treatment. But the white blood cell (WBC) kept increasing out of control. The patient refused the following treatment and discharged on day 25.

**Figure 3:**
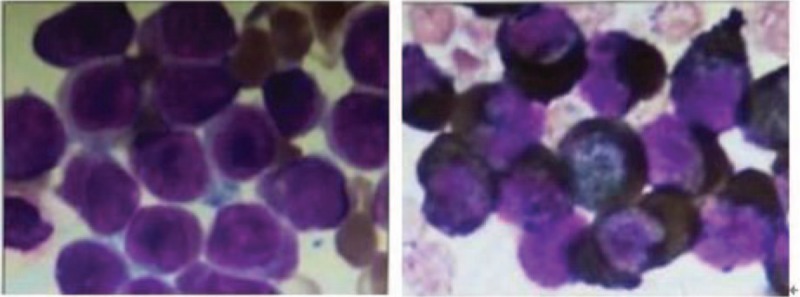
Bone marrow of case C(PLZF/RARα).

**Figure 4:**
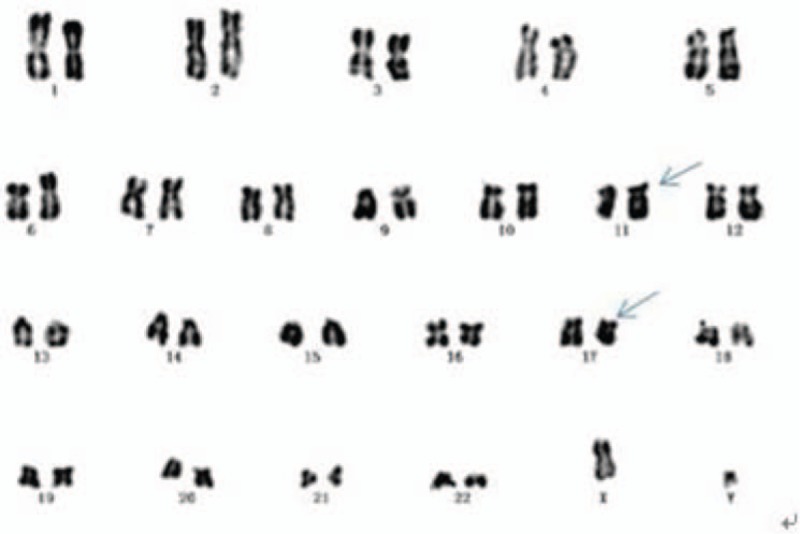
Karyotype of case C 46, XY? t(11,17)(q23,q21)/46,XY.

**Figure 5:**
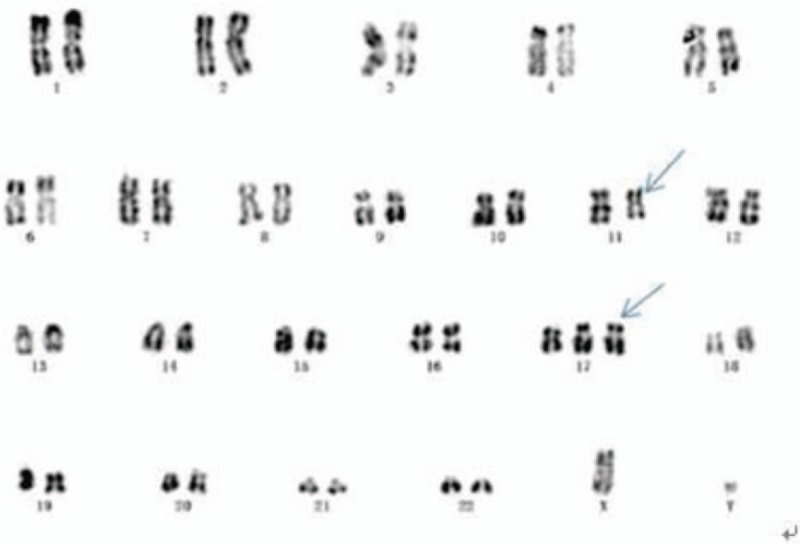
Karyotype of case C 46, XY?11q-,17q+/47,idem,+17q+.

*Case B* A 44 years old male suffered pain from lower limbs and hip. Abnormal CBC informed a high WBC (52.07 × 10^9^/L), anemia (82 g/L) and thrombocytopenia (41 × 10^9^/L). Bone marrow aspirate suggested an abnormal promyelocyte of 93.6% indicating APL (Fig. 3). Flow cytometry was performed to confirm the diagnosis. Result of karyotype analysis showed 46, XY,?t(11,17)(q23,q21)/46, XY (Fig. 4).PLZF-RARα was positive using RT-PCR detection. So ATO and chemotherapy with DA (daunorubicin 45 mg/m^2^/d for 3 days and cytarabine 100 mg/m^2^/d for 7 days) regimen were given at the same time. Four months later, the patient was admitted to hospital again for further management.BM aspirate still revealed predominant blasts. Chromosome analysis showed 46, XY,?11q-,17q+/47, idem,+17q+ (Fig. 5). The coagulation got worse and finally he got the disseminated intravascular coagulation (DIC).

*Case C* 52-year-old male patient presented with fever for 3 weeks invalid to antibiotics and gingival bleeding for 1 week.CBC test indicated anemia and thrombocytopenia. Abnormal coagulation index showed the prothrombin time (PT) of 20.7 seconds, the fibrinogen of 0.6 g/L. Proportion of promyelocytes in the bone marrow was 19.6%, and the flow cytometry indicated a positive of CD33, CD117, CD13, CD123, CD9, CD64, MPO, and CD15. RT-PCR and chromosome analysis described a fuse gene of PLZF-RARα and 47, XY,+8/ 47, idem, t(11,17)(q23,q21)/46, XY. ATRA and ATO were used immediately. Subsequently, chemotherapy was added with DA regimen. He suffered hemoptysis, heart failure, and septicemia of methicillin-resistant staphylococcus aureus during the period of myelosuppression. In the following consolidation treatment, the patient received 3 courses of ATRA and CAG combination treatment (cytarabine 20 mg/12 hours for 14 days, aclarubicin 20 mg/d for 4 days, granulocyte stimulating factor 400 mg for 14 days). Then he got a complete remission (CR) without PLZF-RARα detected. Medium-dose cytarabine with daunorubicin were given (cytarabine 2000 mg per 12 hours for 3 days and daunorubicin 60 mg on the first day). The following treatment regimen was still under discussion. So far, the patient has survived for 7 months and remains CR.

The clinical characteristics of the 3 patients have been shown in Table 1.

**Table 1:**
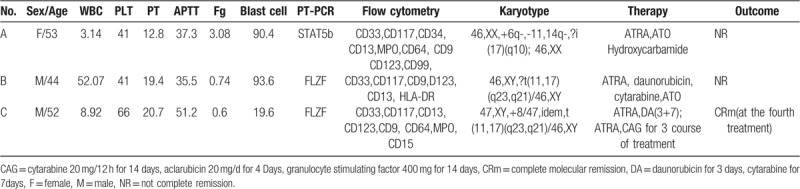
The clinical characteristics of the 4 patients.

## 3 Literature review and discussion

According to the prior report, *STAT5b-RARα* fusion gene occurs as a result of an interstitial deletion within chromosome 17(STAT5b) and RARα.^[8]^ Unlike PML-RARα and PLZF-RARα, STAT5b-RARα singly heterodimerized with RARα, so there is no *RARα/STAT5b* fusion gene, whereas PML-RARα and PLZF-RARα have both single and multimeric complexes (RARα-PML and RARα-PLZF). STAT5b belongs to a family of latent cytosolic transcription factors which were activated by Janus kinase (JAK) tyrosine kinases.^[11]^ STAT5b targets genes relevant to hematopoiesis include c-myc and IL2R, which can induce the expression of anti-apoptotic gene bcl-x, and inhibit the apoptosis of myeloid cells in the terminal differentiation. Meanwhile, it can activate P13 kinase/AKT signal transduction pathway to promote cell growth.^[12]^ STAT5b-RARα can enhance the activity of STAT3, contribute to leukemogenesis by interaction with the STAT3 oncogene pathway.^[12]^ The precise diagnosis of *STAT5b-RARα* fusion gene needs not only RT-PCR, but also fluorescence in situ hybridization(FISH) and next-generation sequencing (NGS).^[11,13]^

Since there are only 12 cases (including this one) of STAT5b-RARα-positive leukemia APL been reported (Table 2), the epidemiological data was still uncertain. The median age of the 12 patients was 42.25(17–67) years, and only 2 of the 12 patients were female (Table 2). However, there is no difference of sex distribution in APL with *PML-RARα* fusion gene. As summarized in Table 2, all the 12 patients were treated with ATRA at the first time, and 6 patients combination with ATO. But none of them got CR simply with ATRA and ATO. So the patients with the STAT5b-RARα fusion transcript appear to be resistant to both ATRA and ATO. In combination with IA(Idarubicin and cytarabine), DA, FLA (fludarabine and cytarabine), FLAG (fludarabine, cytarabine and granulocyte stimulating factor), and mitoxantrone some patients got complete remission (CR) (cases 3, 5, 7, 9, 10).^[13–15]^ But they might relapse in a short time. In case7 the patient got CR after the first induction therapy with DA, and relapsed at the 9th month. In cases 3, 7, 10, the patients were treated with hematopoietic stem cell transplantation (HSCT), they had prolonged the overall survival (OS), but some of them died because of the complication related with HSCT. In case 5, the patient got CR with polychemotherapy, but on the 41th month he got myelodysplastic syndrome. On the 75th the STAT5b-RARα recurred and finally died of pneumorrhagia. In case 11,^[16]^ after 2 unsuccessful courses with ATRA, ATO, mitoxantrone and IA, decitabine and AA (aclacimomycin and cytarabine)/IA combination therapy (decitabine 25 mg/d for 3 days in every course, decitabine +AA for 3 courses, and decitabine+ IA for 3 courses) was given. The patient achieved a CR after the first course of decitabine treatment and STAT5b-RARα fusion transcript changed to be negative at last. After 1 year of follow-up, the patient remains in CR.^[16]^ In our case, ATRA and DA was given in the induction treatment, Case 4 got CR after taking the chemotherapy of CAG regimen.

**Table 2:**
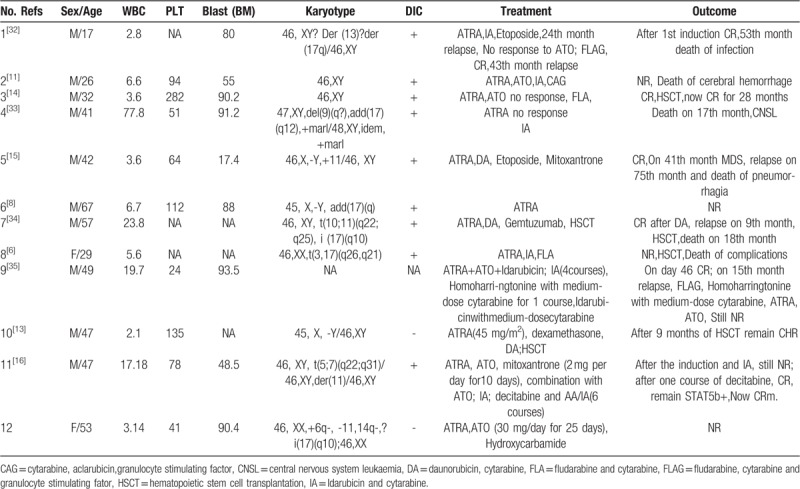
Characteristics of STAT5b/RARa APL patients.

The promyelocytic leukemia zinc-finger (PLZF) gene was initially identified by its rearrangement in an APL with t (11; 17) (q23; q21). PLZF-RARα-positive APL is the most common atypical APL (0.8%), the chromosomal translocations occurs within chromosome 11 and 17, leading to *PLZF-RARα,* and *RARα-PLZF* fusion gene.^[17,18]^*PLZF* can raise many transcriptional auxiliary inhibitors through the POZ domain, such as mSin3A, N-CoR, SMRT, and HDAC.^[18,19]^ At the same time it can make combination with transcription auxiliary inhibitor ETO, so as to inhibit the transcription of target genes. Although the appearance of t (11; 17) on the cytogenetic level in acute myeloid leukemia are identical, but they are diverse on the molecular level. There is a report which describes five different fusion genes: *MLL-LASP1*, *MLL-MLLT6/AF17*, *MLL-ACACA* or *MLL-SEPT9/MSF*, and *PLZF-RARα*, involving the long arms of chromosomes 11(q23) and 17(q12-25).^[20,21]^ So appropriate molecular analysis and cytogenetics are essential.

Here are 21 cases of PLZF-RARα-positive APL (Table 3), the median age is 50 (23–83) years old. They had a poor response to ATO and ATRA. Some patients might got CR and prolonged survival after undergoing intensive chemotherapy including DA, IA, or medium-dose of cytarabine. But most of them relapse in a short time. Case i undertook ATRA, DA(daunorubicin for 3 days and cytarabine for 7 days) and 3 courses of CAG, and achieved CR at last. In case f^[22]^ the patient got CR with DA and medium-dose of cytarabine. In case r^[23]^ the patient was 83 years old, with the therapy of ATRA and daunorubicin for 3 courses, complete histological response (CHR) was achieved and survived for more than 24 months. In case j, n, o, p,^[10,17,24]^ they had allo-HSCT and the OS had prolonged remarkably. Interestingly, RT-PCR confirmed *PLZF-RARα* fusion gene in all the three cases, but not all of PLZF-RARα-positive patients have the chromosome change of t(11; 17) (q23; q21).^[6,10,36]^ RT-PCR confirmed formation of a *PLZF/RARa* fusion gene in all the 3 cases, so the reason needs more researches and the accurate diagnosis should be made by complex means.^[25]^

**Table 3:**
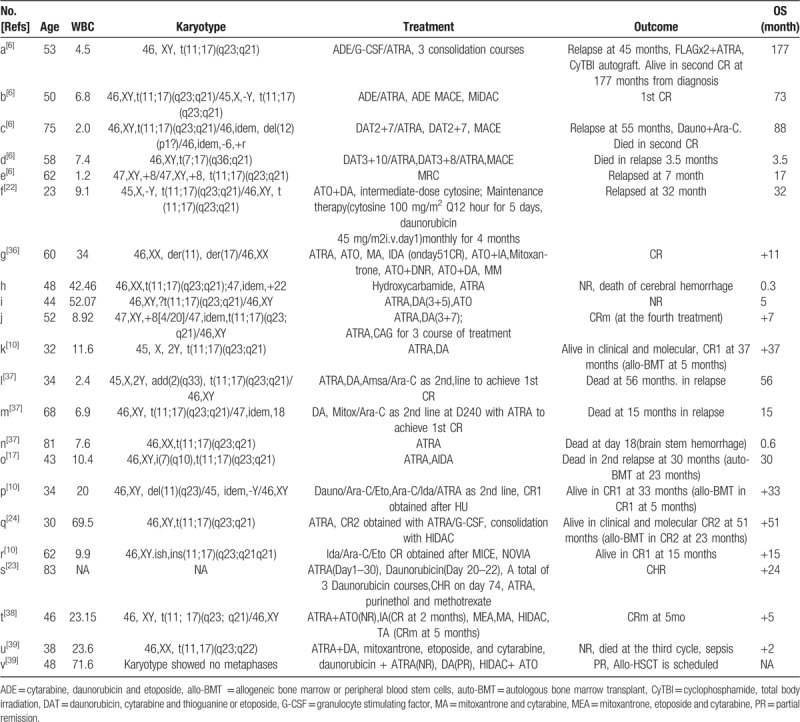
The clinical characteristics of the PLZF/RARa patients.

From prior point of view, since the karyotype of t(11;17) can strongly block differentiation, the PLZF-RARα-positive APL is characterized by poor response to ATO.^[26]^ However, some studies suggest that this subtype of APL is not completely resistant to all differentiation approaches and may be response to ATRA,^[27,28]^ which can induce the persistent deregulation of cell cycle. In a number of views, PLZF-RARα has a relative good response to combined chemotherapy which is used in acute myeloid leukemia.^[10,18,28]^ And different from typical APL (PML-RARα), if there is a suitable donor in patients with t(11;17),it seems reasonable to consider allo-HSCT in first CR. Even if they are not suitable for HSCT, clinical trial should be considered.^[29]^

## 4 Conclusions

Neither PLZF-RARα-positive nor STAT5b-RARα-positive APL is sensitive to ATRA and ATO. Combined chemotherapy should be considered first when a patient is diagnosed with PLZF-RARα-positive APL. However, there is no standard or recommended protocols for STAT5b-RARα-positive APL until now. Since the prognosis and long-term outcome of STAT5b-RARα-positive APL are poor, more clinical trials and researches should be taken. Decitabine combination chemotherapy, HSCT and targeted therapy should be considered, however, it is still unknown whether this regimen will be effective in the future. Other kinds of atypical APL such as NuMA-RARα, NPM-RARα, F1P1L1-RARα, BCOR-RARα and PRKAR1A-RARα positive APL are proved to be effective to ATRA and ATO.^[30,31]^ In a word, although atypical APL is rare, it remains a challenge to all of us.

## Acknowledgments

The patients have provided informed consent for publication of the cases.

## Author contributions

**Data curation:** Jing Wang.

**Formal analysis:** Xiao-Xue Wang, Li-Jun Zhang.

**Resources:** Jing Wang.

**Visualization:** Li-Jun Zhang.

**Writing – original draft:** Xiao-Xue Wang, Jing Wang.

**Writing – review & editing:** Li-Jun Zhang.
